# MoS_2_-Carbon Inter-overlapped Structures as Effective Electrocatalysts for the Hydrogen Evolution Reaction

**DOI:** 10.3390/nano10071389

**Published:** 2020-07-17

**Authors:** Po-Chia Huang, Chia-Ling Wu, Sanjaya Brahma, Muhammad Omar Shaikh, Jow-Lay Huang, Jey-Jau Lee, Sheng-Chang Wang

**Affiliations:** 1 X-ray Scattering Group, National Synchrotron Radiation Research Center, Hsinchu 300, Taiwan; huang.pc@nsrrc.org.tw (P.-C.H.); jjlee@nsrrc.org.tw (J.-J.L.); 2 Department of Materials Science and Engineering, National Cheng Kung University, Tainan 701, Taiwan; chialingwu20@gmail.com (C.-L.W.); sanjayaphysics@gmail.com (S.B.); JLH888@mail.ncku.edu.tw (J.-L.H.); 3 Institute of Medical Science and Technology, National Sun Yat-sen University, Kaohsiung 804, Taiwan; omar.offgridsolutions@gmail.com; 4 Department of Mechanical Engineering, Southern Taiwan University of Science and Technology, Tainan 710, Taiwan

**Keywords:** MoS_2_-carbon, layer-expanded, hot-injection method, electrocatalysis

## Abstract

The ability to generate hydrogen in an economic and sustainable manner is critical to the realization of a future hydrogen economy. Electrocatalytic water splitting into molecular hydrogen using the hydrogen evolution reaction (HER) provides a viable option for hydrogen generation. Consequently, advanced non-precious metal based electrocatalysts that promote HER and reduce the overpotential are being widely researched. Here, we report on the development of MoS_2_-carbon inter-overlapped structures and their applicability for enhancing electrocatalytic HER. These structures were synthesized by a facile hot-injection method using ammonium tetrathiomolybdate ((NH_4_)_2_MoS_4_) as the precursor and oleylamine (OLA) as the solvent, followed by a carbonization step. During the synthesis protocol, OLA not only plays the role of a reacting solvent but also acts as an intercalating agent which enlarges the interlayer spacing of MoS_2_ to form OLA-protected monolayer MoS_2_. After the carbonization step, the crystallinity improves substantially, and OLA can be completely converted into carbon, thus forming an inter-overlapped superstructure, as characterized in detail using X-ray diffraction (XRD), Fourier transform infrared spectroscopy (FTIR), Raman spectroscopy, transmission electron microscopy (TEM) and X-ray photoelectron spectroscopy (XPS). A Tafel slope of 118 mV/dec is obtained for the monolayer MoS_2_-carbon superstructure, which shows a significant improvement, as compared to the 202 mV/dec observed for OLA-protected monolayer MoS_2_. The enhanced HER performance is attributed to the improved conductivity along the c-axis due to the presence of carbon and the abundance of active sites due to the interlayer expansion of the monolayer MoS_2_ by OLA.

## 1. Introduction

The ability to be an energy carrier with an extremely high gravimetric energy density (142 MJ kg^−1^) makes molecular hydrogen (H_2_) one of the most promising candidates for storing renewable energy in the form of chemical bonds [1,2]. Compared to hydrogen evolution from methane (CH_4_(g) + 2H_2_O(g) → CO_2_(g) + 4H_2_(g)), electrocatalytic water splitting (2H_2_O(l) → 2H_2_(g) + O_2_(g)) is a more sustainable and environmentally friendly alternative [3]. However, the hydrogen evolution reaction (HER) requires the use of an electrocatalyst to lower the activation energy and overpotential needed to split water. While noble metals like platinum (Pt) are known to be highly efficient catalysts for HER, they are not sustainable because of their high price and scarcity [4].

Recently, two-dimensional (2D) materials have emerged as promising candidates for high-performance electrocatalytic applications due to their unique chemical, physical and electronic properties [5]. Among these, MoS_2_ has been widely researched as an electrocatalyst for HER due to its attractive properties like presence of abundant catalytically active sites [6], lower surface defects [7] and improved carrier mobility, resulting in high exchange current density [8]. Single-layer MoS_2_ (1T-MoS_2_) in particular has unique metallic-like active edges that can improve catalytic activity by enhancing both the electron transport and ion diffusion [9,10,11,12,13]. Electron transport is significantly enhanced due to its distorted octahedral coordination, while ion diffusion is improved due its hydrophilic nature owing to the ~1 nm wide diffusion channels formed between 1T-MoS_2_ layers [13,14]. According to a Nørskov et al. [15] study, when the MoS_2_ layer expands every 0.37 Å, the ΔGH* would reduce 0.05 eV, and this leads to MoS_2_ having catalytic properties similar to Pt (0 eV). Nevertheless, since 1T-MoS_2_ is a thermodynamically metastable phase, it can easily undergo an irreversible transition to the more stable 2H-MoS_2_ phase due to the van der Waals interaction [9,16,17]. In order to solve this issue, suitable dopants like Re, Mn and Tc or interlayer insertion of guest ions like Na^+^ and NH^4+^ that possess electron donor capabilities need to be utilized to stabilize the 1T-MoS_2_ phase [8,12,18,19].

Consequently, 1T-MoS_2_ nanosheets have attracted significant attention in the research community due to their outstanding performance in energy storage devices [7,10,11,18,20], electrocatalysis [8,21,22,23,24], photoelectrochemical cells [14,22,25,26] and field-effect transistors [27,28], among others. Additionally, integrating 1T-MoS_2_ with interlayer carbonaceous materials like grapheme [21,27], carbon nanotubes (CNTs) [29,30], reduced graphene oxide (RGO) [31], N-doped carbon or spherical carbon via anchoring [32], growth [33], loading or dispersion can further increase electrical conductivity, structural integrity and electrocatalytic activity [30,34].

Oh et al. [8] used the solvothermal process for the synthesis of rose-like MoS_2_ nanostructures with an expanded interlayer spacing of 0.99 nm, which had more active edge sites with lower activation energy for the electrochemical HER. The rose-like MoS_2_ delivered better HER catalytic properties, and the Tafel slope was 50 mV/dec. Li et al. [18] also fabricated MoS_2_-carbon interlayer structures by using Na_2_MoO_4_, thiourea, oleic acid (OA), surfactant (P123) and dopamine for amination, followed by annealing at 850 °C. After carbonization, the MoS_2_ layer expanded to 0.98 nm, which facilitated the Li-ion diffusion at the interface, demonstrating more prominent capacity to adsorb Li atoms. However, the synthesis process used environmentally unfriendly chemicals. Therefore, we would like to promote a relatively green and facile process for the synthesis of MoS_2_/carbon electrocatalysts.

This study is an extension of our previous report [23]. In this study, we used a different Mo precursor and utilized a facile hot-injection method to synthesize single-layer MoS_2_-carbon inter-overlapped structures that show promise as electrocatalysts for HER. Different from our previous report, besides being used as the solvent, oleylamine (OLA) also played the role of the capping surfactant to protect the synthesized monolayer 1-T MoS_2_ and enabled interlayer expansion. Next, a simple carbonization step via addition of tributylphosphine (TBP) followed by annealing was utilized to transform the capped OLA into crystalline carbon. The proposed single-layer MoS_2_-carbon inter-overlapped superstructure demonstrated excellent HER performance with a lower onset potential, higher current density and improved Tafel slope, as compared to OLA-protected monolayer MoS_2_ before carbonization.

## 2. Materials and Methods

### 2.1. Preparation of OLA-Protected Monolayer MoS_2_

The Mo precursor was prepared by dispersing 1 mmol of MoO_3_ (Molybdenum trioxide, Alfa Aesar, 99.998, Heysham, England) in 5 mL NH_4_OH (Ammonia solution, SHOWA, 28%, Osaka, Japan) and heated to 80 °C in Ar atmosphere until the mixture became transparent, followed by the addition of 10 mL OLA (Oleylamine, ACROS, 90%, Geel, Belgium). To prepare the S precursor, 4 mmol of S powder (sulfur powder, Sigma Aldrich, 98%, St. Louis, MO, USA) was dispersed in 10 mL of OLAand heated to 155 °C followed by cooling down to 80 °C. Next, the S-precursor was injected into the Mo-precursor and kept at 80 °C until the solution turned a clear red color. The solution was then heated to 280 °C, the heating was continued for 2 h to form the MoO_x_ complex crystal phase, and the temperature was further raised to 350 °C for 1 h to enable phase transformation to MoS_2_, as described in further detail in our previous study [23]. The final products were washed by sonication using 20 mL hexane (J.T. Baker, 95%) followed by centrifugation at 6000 rpm using 5 mL acetone (J.T. Baker, >99.3%) and 5 mL ethanol (J.T. Baker, 96%) for several times. The prepared OLA-protected monolayer MoS_2_ nanopowder samples were finally dried overnight at 60 °C in the oven.

### 2.2. Preparation of MoS_2_-Carbon Inter-Overlapped Structure

The OLA-protected monolayer MoS_2_ was mixed with 3 mL of TBP (tributylphosphine, Kanto Chemical Co., Ltd.,98%, Tokyo, Japan) and 100 mL of deionizer (DI) water and stirred overnight in air to enable self-polymerization. Next, the carbonization step was performed by annealing at 850 °C for 2 h at low vacuum in a quartz tube within argon, followed by cooling down to room temperature to obtain the MoS_2_-carbon inter-overlapped structure.

### 2.3. Characterization

X-ray diffraction (XRD) analyses were performed using synchrotron radiation (Beamline 17A, National Synchrotron Radiation Research Center, Hsinchu, Taiwan) operating an electron energy of 1.5 GeV, current of 300 mA and wavelength of 1.3214 nm. The 2D diffraction signals were collected by Mar345 image plate detector (marXperts, Norderstedt, Germany) for 600 s followed by conversion to one-dimensional patterns using GSAS-II software [35]. Microstructural analysis was performed by transmission electron microscopy (Tecnai G2 F20 FEG-TEM, Amsterdam, The Netherlands) using an ultrahigh-resolution analytical electron microscope (UHRFE-SEM, Zeiss, Auriga, Oberkochen, Germany) operating at an acceleration voltage of 200 kV. X-ray photoelectron spectroscopy (XPS, PHI-5000, Waltham, MA, USA) measurements were performed by using Mg Kα = 1253.6 eV excitation source. The binding energies obtained during the XPS spectral analysis were corrected for specimen charging by referencing C 1 s to 284.5 eV. Fourier-transform infrared spectroscopy (FTIR) and Raman spectroscopy were measured at room temperature using a Jasco 460 and Renishaw (514 nm laser), respectively.

### 2.4. Electrochemical Measurement

Electrochemical measurements were performed using a conventional three-electrode system. Five milligrams of sample and 40 μL Nafion solution (Sigma Aldrich, 5%, St. Louis, MO, USA)) (5 wt. %) were dispersed in 1 mL water/ethanol solution by sonicating for 1 h to form a homogeneous ink. Then, 4 μL of the ink (containing 20 μg of the catalyst) was loaded onto a glassy carbon electrode with 3 mm diameter (loading ca. 0.285 mg cm^−2^). Linear sweep voltammetry with a scan rate of 5 mV/s^−1^ was conducted in 0.5 M H_2_SO_4_ (purged with pure N_2_) using modified glassy carbon as the working electrode, Pt as the counter electrode and Ag/AgCl as the reference electrode. All the potentials were calibrated to a reversible hydrogen electrode (RHE) in 0.5 M H_2_SO_4_, where E (RHE) = E (Ag/AgCl) + 0.237 V.

## 3. Results and Discussion

### 3.1. Phase and Chemical Bonding Analyses

**Figure 1:**
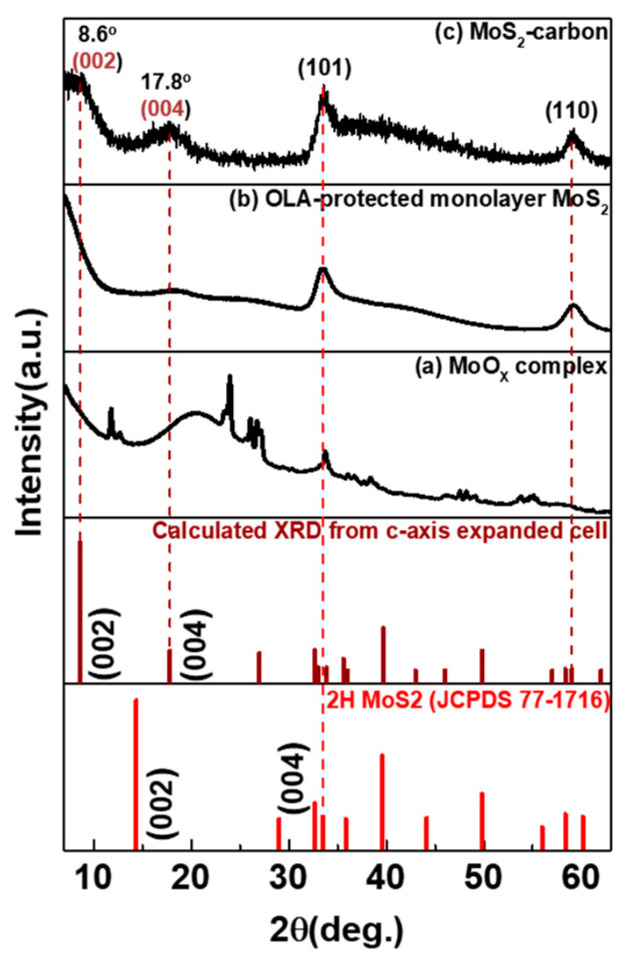
XRD spectra of as-prepared samples at different reaction temperatures and after the carbonization step. (**a**) MoO_x_ complex, (**b**) oleylamine (OLA)-protected monolayer MoS_2_ and (**c**) MoS_2_-carbon.

The XRD spectra for as-prepared samples at different reaction temperatures and after the carbonization step are shown in Figure 1. After hot-injection of the S-precursor and the reaction temperature being held at 280 °C, several peaks related to MoO_x_ complex (mixture of MoO_3_, Mo_8_O_23_, Mo_4_O_11_ and MoO_2_ [23]) were observed in the XRD spectra, in Figure 1a, thus indicating that the temperature was not sufficient for the formation of MoS_2_. When the temperature was further increased to 350 °C, which is closer to the boiling point of OLA, the reaction was under an intense environment that could trigger MoO_x_ recrystallization to monolayer MoS_2_. The presence of a relatively broader diffraction peak as shown in Figure 1a indicates poor crystallinity of the monolayer MoS_2_. In order to enhance the crystallinity of monolayer MoS_2_, we further mixed with TBP and annealed at 850 °C for 2 h under low vacuum in a quartz tube to enable carbonization. As seen in Figure 1c, the XRD spectra indicated that the diffraction peaks corresponding to (002), (101) and (110) planes were significantly narrower than the non-annealed sample. Furthermore, a new diffraction peak of 1T-MoS_2_ (002) was observed at a 2θ value of 8.6° [8], which shifted from its conventional value of 8.9°. This can be explained using Bragg’s Law as the lattice d spacing experiences a distortion from 0.99 Å (8.9°) to 1.02 Å (8.6°) due to the formation of the MoS_2_-carbon inter-overlapped superstructure. Further analysis will be performed in future works to explain the reason for the peak shift in greater detail.

**Figure 2:**
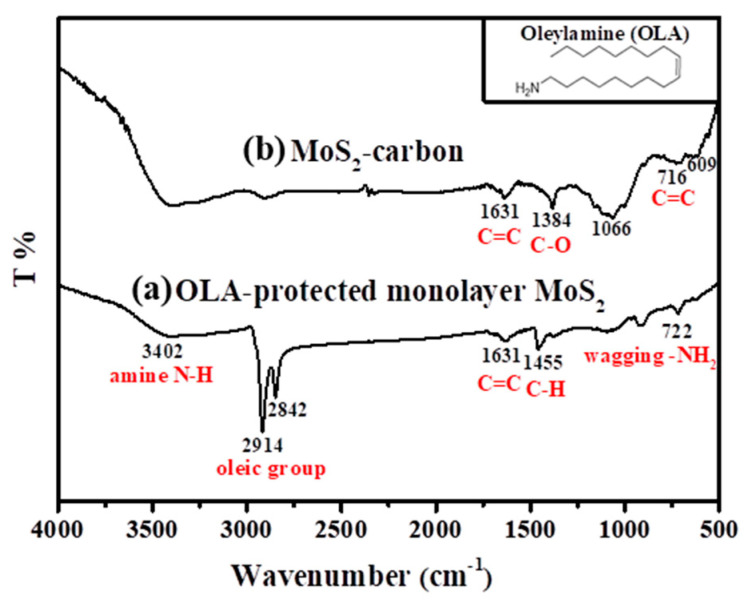
FT-IR spectra of (**a**) OLA-protected monolayer MoS_2_ and (**b**) MoS_2_-carbon. Insert of the figure is the molecular structural formula of OLA.

The FTIR spectra of the OLA-protected monolayer MoS_2_ compared to the MoS_2_-carbon inter-overlapped structure is shown in Figure 2, and the monolayer MoS_2_ (before carbonization) showed the presence of characteristic peaks corresponding to the presence of OLA. These included NH_2_ (722 cm^−1^), C–H (1455 cm^−1^), oily groups (CH_2_ at 2842 cm^−1^ and CH_3_ at 2914 cm^−1^) [36,37], amine (N–H 3402 cm^−1^) and C=C (1631cm^−1^) [20]. However, after carbonization, only the C=C remained, while all the other functional groups were removed, as shown in Figure 2b. The other peaks of MoS_2_/carbon at 716 cm^−1^, 1384 cm^−1^ and 1631 cm^−1^ [38] corresponded to functional groups of carbon (C–C, C–O, C=C) whereas the peaks at 609 cm^−1^ and 1066 cm^−1^ corresponded to MoS_2_ [39]. Consequently, it was concluded that the high-temperature carbonization treatment effectively eliminated OLA and transformed it completely into elemental carbon.

**Figure 3:**
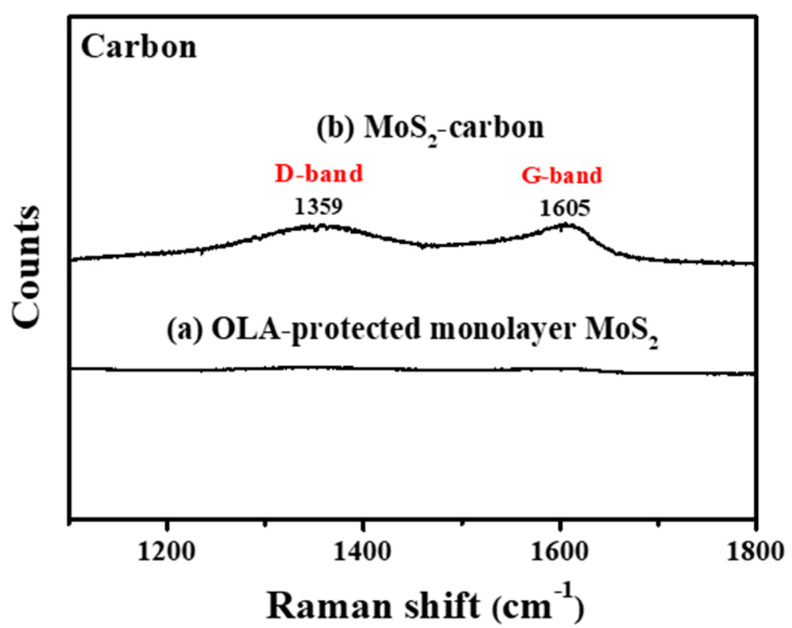
Raman spectroscopy of (**a**) OLA-protected monolayer MoS_2_ and (**b**) MoS_2_-carbon.

Figure 3 clearly shows Raman shift from 1100 to 1800 cm^−1^ before and after carbonization. It is observed that, after carbonization, there were significant peaks at 1359 cm^−1^ and 1597 cm^−1^ which correspond to graphite (Defective carbon, D-band and Ordered carbon, G-band), respectively [20,40], thus proving the successful carbonization of OLA. The D-band originates in sp^3^ hybridized carbon where the peak intensity count reveals the number of defects. G-band carbon atoms are sp^2^ hybridized and represent the common layered structure of graphite. The peak intensity ratio of the I_D_/I_G_ band is usually used to infer the degree of defects present and the conductivity of the material. If the I_D_/I_G_ band ratio is higher than 1 then the sp^3^ hybridization is dominant, and the carbon has relatively more defects and lower conductivity. On the contrary, if the I_D_/I_G_ band ratio is less than 1, the carbon is less defective and has improved conductivity [41,42]. In this study, the I_D_/I_G_ ratio was calculated to be 0.88, which reveals that the carbon obtained via carbonization of OLA between the MoS_2_ layers consisted mostly of an ordered graphitic structure with relatively fewer defects and improved conductivity, thus enabling increased electrochemical reaction activity. Based on the results obtained from FTIR and Raman spectroscopy, it can be concluded that the carbonization treatment protocol can successfully transform the OLA completely into elemental carbon.

### 3.2. Microstructure Analysis

**Figure 4:**
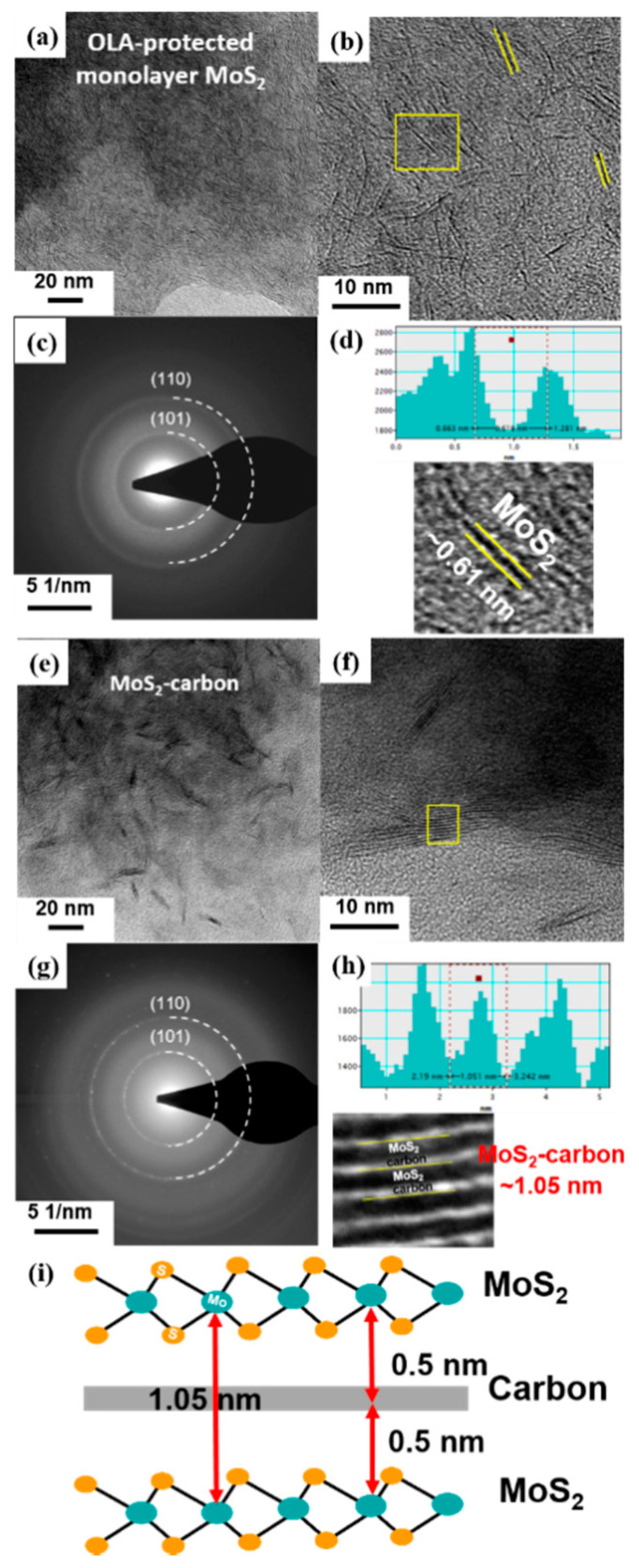
TEM images, HRTEM images with SADP and calibration plot for measuring the lattice spacing of (**a**–**d**) OLA-protected monolayer MoS_2_ and (**e**–**h**) MoS_2_-carbon. (**i**) Schematic illustration of the MoS_2_-carbon inter-overlapped structure.

The TEM images and schematic illustrations of MoS_2_ before (Figure 4a–d) and after (Figure 4e–i) carbonization are shown in Figure 4. It was observed that the MoS_2_ crystals had a random distribution before carbonization, as shown in Figure 4a,b. Furthermore, the select-area diffraction pattern (SADP) shown in Figure 4c demonstrates that the monolayer MoS_2_ had a polycrystalline structure with relatively poor crystallinity. The two diffraction rings observed correspond to the (101) and (110) planes of monolayer MoS_2_, respectively. The high-resolution TEM (HRTEM) image revealed a lattice spacing of about 0.61 Å, which corresponds to the (002) plane [40] of single-layer MoS_2_ (Figure 4d).

After carbonization, the TEM and HRTEM images of the obtained MoS_2_-carbon inter-overlapped structures are shown in Figure 4e,f. It was clearly observed that the carbonization process resulted in ordered stacking of the MoS_2_ crystals due to the presence of the interlayer carbon. Furthermore, the SADP shown in Figure 4g displays a relatively smaller light-spot on the diffraction ring, thus revealing the improved crystallinity observed after carbonization. The two diffraction rings also correspond to the (101) and (110) planes of MoS_2_, respectively. The partially magnified HRTEM image in Figure 4h and the calibration plot in Figure 4f reveal that the lattice spacing of MoS_2_ after carbonization expanded to 1.05 nm, which was larger than that observed before carbonization. These results can be attributed to the insertion of the interlayer carbon between the MoS_2_ layers. The schematic illustration of the MoS_2_-carbon inter-overlapped superstructure is shown in Figure 4i. During monolayer MoS_2_ transformation to MoS_2_-carbon inter-overlapped superstructure, the lattice expanded 0.44 nm, from 0.61 to 1.05 nm. Consequently, the reduced ΔG_H_* and increased exposure of active sites resulted in improved catalytic performance.

### 3.3. Elemental Composition and Valence State

**Figure 5:**
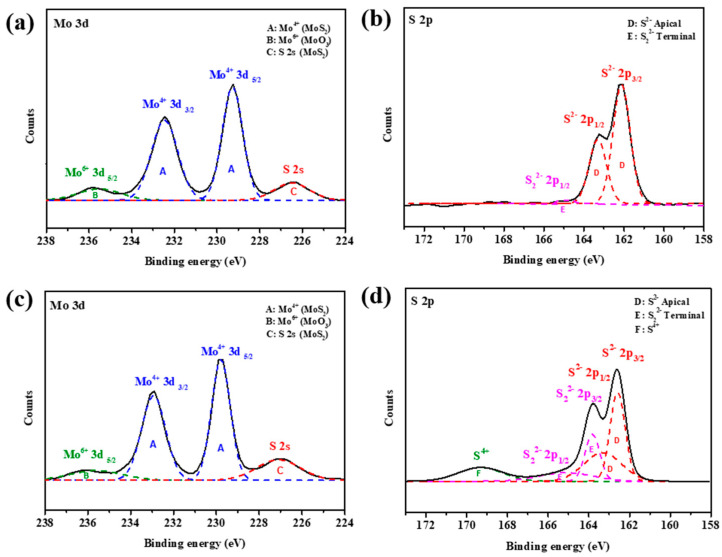
XPS analysis of Mo 3d and S 2p spectra of (**a**,**b**) OLA-protected monolayer and (**c**,**d**) MoS_2_-carbon.

The Mo and S valence states of OLA-protected monolayer MoS_2_ and MoS_2_-carbon inter-overlapped structures were characterized by X-ray photoelectron spectroscopy (XPS) as shown in Figure 5. The XPS spectra Mo and S valence states of OLA-protected monolayer MoS_2_, as shown in Figure 5a,b, can be deconvoluted into four peaks. The two main intense peaks at binding energies of 229.2 and 232.5 eV corresponded to Mo^4+^ 3d_5/2_ and Mo^4+^ 3d_3/2_ of MoS_2_ (symbol A) [22], respectively. The broad weaker peak at a binding energy of 235.8 eV corresponded to Mo^6+^ 3d_5/2_ (symbol B) [17,43], which can be attributed to environmental oxidation or absorption of oxygen on the surface. The peak observed at a binding energy of 226.2 eV corresponded to S 2s orbital of MoS_2_ (symbol C) [44]. The S 2p spectrum was obtained using high-resolution XPS as shown in Figure 5b. The main doublet peaks at binding energies of 161.6 and 162.8 eV corresponded to the S 2p_3/2_ and S 2p_1/2_ of MoS_2_ (symbol D) [40,45], respectively. In addition, the weak peak at a binding energy of 165 eV corresponded to S_2_^2−^ 2p_1/2_ (symbol E) [46], which suggests the existence of apical S-defects that can improve the electrocatalytic activity towards HER.

After carbonization, the Mo valence states deconvoluted into four peaks that were similar to the OLA capped monolayer MoS_2_. However, there was a significant increase in the intensity of the S valence state deconvoluted peaks of S_2_^2−^ 2p_3/2_ and S_2_^2−^ 2p_1/2_. Furthermore, a new peak was observed for a binding energy of 169.2 eV, which can be attributed to S^4+^ species (symbol F) [47]. The presence of S^4+^ and S_2_^2−^ defects present in MoS_2_ suggested that HER performance should be enhanced after carbonization. In particular, recent studies have demonstrated that the presence of lattice defects like S vacancies can enable activation of the inert MoS_2_ basal plane. Hong et al. [48] extensively studied point defects in 2D MoS_2_ and found that the S vacancy is a highly energy-favorable defect present on the basal plane. Li et al. [49] researched different active sites in MoS_2_ and concluded that S vacancies contribute to the catalytic activity and act as active sites for HER in addition to exposed edges. Furthermore, Li et al. [50] reported that introduction of S vacancies and lattice strain can yield an optimal hydrogen adsorption free energy (∆G_H_) equal to 0 eV, thus resulting in a high intrinsic HER activity. This is because the S vacancy produces under-coordinated Mo atoms, which introduce gap states that promote hydrogen binding and consequently improve the HER performance.

### 3.4. Electrocatalytic HER Performance

**Figure 6:**
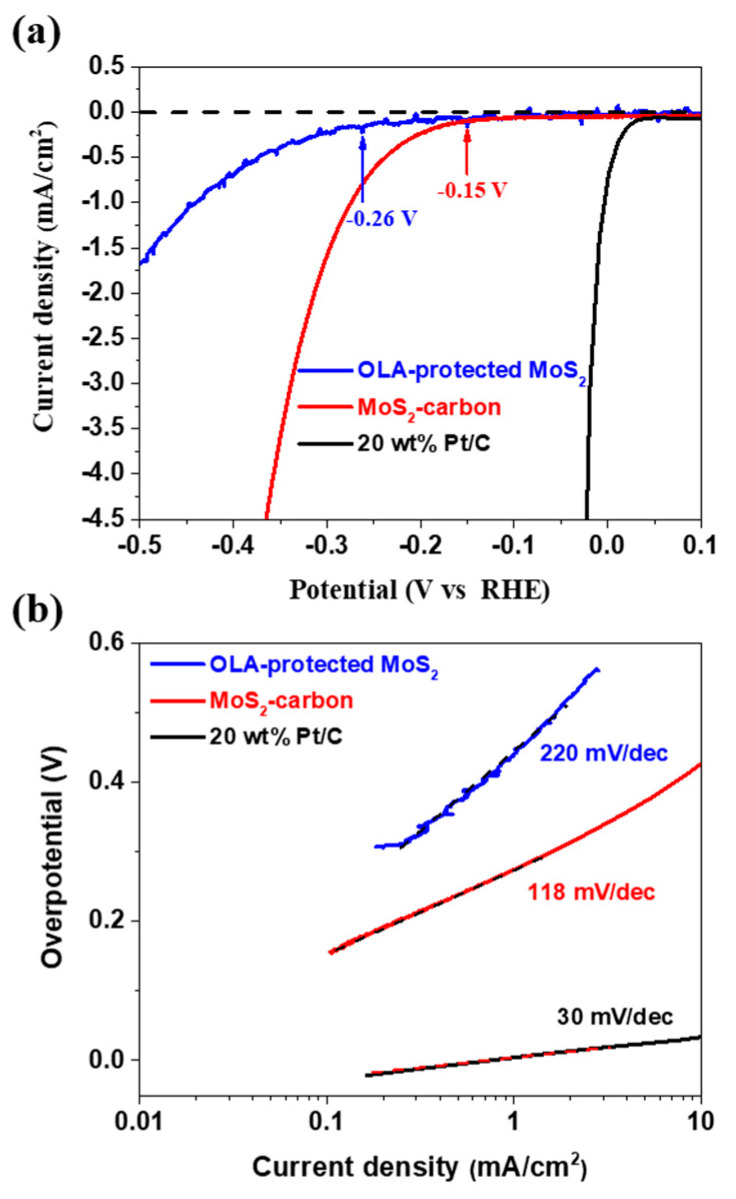
The HER performance of OLA-protected monolayer MoS_2_ (**blue**), MoS_2_-carbon (**red**) and 20 wt% Pt/C. (**a**) The polarization curves and (**b**) the corresponding Tafel plots obtained using a glassy carbon working electrode with a catalyst loading of 0.28 mg/cm^2^ and a scan rate of 5 mV/s.

The electrocatalytic HER performance of OLA-protected monolayer MoS_2_ and MoS_2_-carbon inter-overlapped superstructure was investigated using a conventional three-electrode electrochemical system with 0.5 M H_2_SO_4_ as the electrolyte. The sample was drop-casted on a glassy carbon (GC) working electrode, and Pt wire and Ag/AgCl were used as the counter and reference electrodes, respectively. The polarization curve recorded for MoS_2_-carbon as shown in Figure 6a displays a small onset potential (*η*) of 0.15 V for HER, which is relatively lower as compared to OLA-protected monolayer MoS_2_ (*η* at −0.26 V). The resulting Tafel slopes of the plots were fitted to the Tafel equation *η* = *b* log (*j*/*j_0_*), where *η* is the overpotential, b is the Tafel slope, *j* is the current density, and *j_0_* is the exchange current density. The observed Tafel slope of the MoS_2_-carbon sample was 118 mV/dec as shown in Figure 6b, which demonstrates improved HER performance as compared to the OLA-protected monolayer MoS_2_ (220 mV/dec). The enhanced HER performance of the MoS_2_-carbon can be attributed to the improved electron conductivity due to the presence of the interlayer carbon. Furthermore, the interlayer carbon also resulted in expansion of the lattice and effective separation of the MoS_2_, thus increasing the exposure of active edges which improve the catalytic activity [14,18,51]. Table 1 describes a comparative analysis of the HER performance for MoS_2_/carbon composite electrocatalysts. The MoS_2_/carbon composites, as reported previously, are prepared by using hydrothermal, solvothermal and chemical vapor deposition methods. These methods are energy and time-consuming and use environmentally unfriendly chemical processes. Therefore, we followed a simple and facile hot-injection method for the synthesis of MoS_2_-carbon electrocatalysts. This method is a relatively green and facile process and can significantly reduce the reaction time, as compared with other methods.

**Figure 7:**
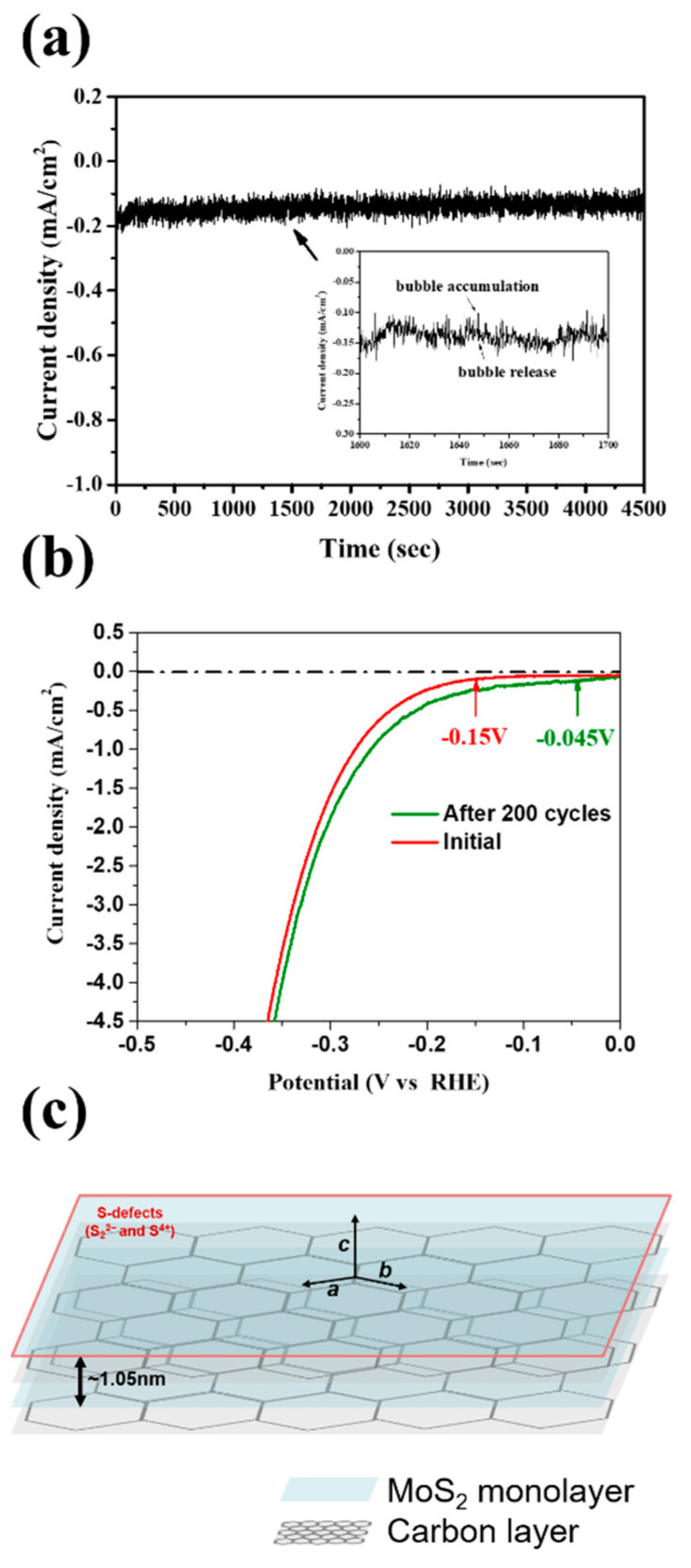
(**a**) Chronoamperometric (j-t) response of the MoS_2_-carbon electrocatalyst for 4500 s at −0.2 V (vs. reversible hydrogen electrode, RHE) which is slightly higher than the onset potential. The inset shows an enlarged image to highlight the bubble accumulation and release. (**b**) LSV testing of the MoS_2_-carbon electrocatalyst for 200 cycles. (**c**) Schematic of the MoS_2_-carbon inter-overlapped superstructure electrocatalyst.

The results of the stability testing for the MoS_2_-carbon electrocatalyst are shown in Figure 7. The chronoamperometric (j-t) response was recorded for 4500 s as shown in Figure 7a. It was observed that the j-t curve had periodic fluctuations, which can be attributed to the H_2_ bubble accumulation and release as shown by the magnified inset in Figure 7a, thus implying that the H^+^ was quickly converted to H_2_. Furthermore, it is interesting to note that the current density showed a gradual increase with time. Therefore, we further performed LSV (linear sweep voltammetry) testing for 200 cycles as shown in Figure 7b. The results agreed with those observed using chronoamperometry and displayed improved catalytic properties after 200 cycles. The onset potential reduced from −0.15 to −0.045 V, while the current density curve also shifted to a lower overpotential. A schematic of the MoS_2_-carbon inter-overlapped structure electrocatalyst is shown in Figure 7c, and the proposed electrocatalyst displayed an improved HER performance that can be attributed to the following reasons: (i) significantly lower onset potential due to the insertion of the interlayer carbon that results in faster electron transfer to the electrolyte; (ii) increased conductivity due to the interlayer carbon that can avoid self-oxidation caused by electron accumulation; (iii) reduction in ΔG_H*_ increases suitability for H^+^ reduction to H_2_ gas; (iv) abundant presence of S^4+^ and S_2_^2−^ defects in the MoS_2_-carbon electrocatalyst enhances the HER catalytic properties.

## 4. Conclusions

In summary, we reported a facile synthesis protocol to obtain MoS_2_-carbon inter-overlapped structures which demonstrated improved electrocatalytic performance for HER. OLA-protected monolayer MoS_2_ was successfully synthesized using hot injection where the OLA not only acted as a solvent but also penetrated and effectively capping surfactant to obtained MoS_2_ monolayers. A simple carbonization protocol was used to transform the OLA into elemental carbon and obtain the MoS_2_-carbon inter-overlapped superstructure. The presence of the interlayer carbon enhanced the conductivity of the electrocatalyst and improved HER performances due to increased exposure of active sites as a result of lattice expansion in the c-axis direction. After carbonization, a number of S-defects (such as S_2_^2−^ and S^4+^) were detected on the MoS_2_-carbon electrocatalyst surface, which further enhanced the HER performance. Furthermore, the MoS_2_-carbon electrocatalyst not only displayed good long-term stability for HER, but it also significantly reduced the onset potential. Given our interesting findings, we anticipate that the proposed MoS_2_-carbon inter-overlapped structure based electrocatalyst will have good practical applicability for HER.

## Figures and Tables

**Table 1:** Comparative analysis of the HER performance of MoS_2_/C electrocatalysts.

Catalysts	Precursor	Onset Potential (V)	Tafel Slope (mV dec^−1^)	Ref.
MoS_2_-WS_2_-CNTs	Na_2_MoO_4_, (C_2_H_5_)_2_NCSSNa·3H_2_O (DEDTC)		50	[8]
MoS_2_/rGO	(NH_4_)_2_MoS_4_, GO, DMF N_2_H_4_·H_2_O	−0.1	41	[52]
N-MoS_2_/C_3_N_4_	DICY, (NH_4_)_2_MoS_4_·4H_2_O, DMF	−0.3	46.8	[53]
MoS_2_/coated CNTs	(NH_4_)_2_MoS_4_, DMF	−0.09	44.6	[54]
MoS_2_/NCNFs	PAN, Na_2_MoO_4_·2H_2_O, graphite rod, DMF	−0.1	48	[55]
MoS_2_/C-cloth	(NH_4_)_2_MoS_4_, carbon cloth	−0.15	50	[56]
MoS_2_/carbon	MoO_3_, NH_4_OH, OLA, TBP	−0.15	118	This work

## References

[1] Wang F.M., Shifa T.A., Zhan X.Y., Huang Y., Liu K.L., Cheng Z.Z., Jiang C., He J. (2015). Recent advances in transition-metal dichalcogenide based nanomaterials for water splitting. Nanoscale.

[2] Gao M.R., Xu Y.F., Jiang J., Yu S.H. (2013). Nanostructured metal chalcogenides: Synthesis, modification, and applications in energy conversion and storage devices. Chem. Soc. Rev..

[3] Lu Q.P., Yu Y.F., Ma Q.L., Chen B., Zhang H. (2016). 2D Transition-Metal-Dichalcogenide-Nanosheet-Based Composites for Photocatalytic and Electrocatalytic Hydrogen Evolution Reactions. Adv. Mater..

[4] Antolini E. (2009). Palladium in fuel cell catalysis. Energy Environ. Sci..

[5] Briggs N., Subramanian S., Lin Z., Li X.F., Zhang X.T., Zhang K.H., Xiao K., Geohegan D., Wallace R., Chen L.Q. (2019). A roadmap for electronic grade 2D materials. 2D Mater..

[6] Anjum M.A.R., Jeong H.Y., Lee M.H., Shin H.S., Lee J.S. (2018). Efficient Hydrogen Evolution Reaction Catalysis in Alkaline Media by All-in-One MoS_2_ with Multifunctional Active Sites. Adv. Mater..

[7] Liu M.M., Zhang C.C., Su J.M., Chen X., Ma T.Y., Huang T., Yu A.S. (2019). Propelling Polysulfide Conversion by Defect-Rich MoS_2_ Nanosheets for High-Performance Lithium-Sulfur Batteries. Acs Appl. Mater. Interfaces.

[8] Thangasamy P., Oh S., Nam S., Oh I.K. (2020). Rose-like MoS_2_ nanostructures with a large interlayer spacing of similar to 9.9 angstrom and exfoliated WS_2_ nanosheets supported on carbon nanotubes for hydrogen evolution reaction. Carbon.

[9] Tsai C., Chan K.R., Norskov J.K., Abild-Pedersen F. (2015). Theoretical insights into the hydrogen evolution activity of layered transition metal dichalcogenides. Surf. Sci..

[10] Wu M.H., Zhan J., Wu K., Li Z., Wang L., Geng B.J., Wang L.J., Pan D.Y. (2017). Metallic 1T MoS_2_ nanosheet arrays vertically grown on activated carbon fiber cloth for enhanced Li-ion storage performance. J. Mater. Chem. A.

[11] Senthil C., Amutha S., Gnanamuthu R., Vediappan K., Lee C.W. (2019). Metallic 1T MoS_2_ overlapped nitrogen-doped carbon superstructures for enhanced sodium-ion storage. Appl Surf. Sci.

[12] Shi S.L., Sun Z.X., Hu Y.H. (2018). Synthesis, stabilization and applications of 2-dimensional 1T metallic MoS_2_. J. Mater. Chem. A.

[13] Geng X.M., Zhang Y.L., Han Y., Li J.X., Yang L., Benamara M., Chen L., Zhu H.L. (2017). Two-Dimensional Water-Coupled Metallic MoS_2_ with Nanochannels for Ultrafast Supercapacitors. Nano Lett..

[14] Liu Q., Li X.L., He Q., Khalil A., Liu D.B., Xiang T., Wu X.J., Song L. (2015). Gram-Scale Aqueous Synthesis of Stable Few-Layered 1T-MoS_2_: Applications for Visible-Light-Driven Photocatalytic Hydrogen Evolution. Small.

[15] Tsai C., Abild-Pedersen F., Nørskov J.K. (2014). Tuning the MoS_2_ edge-site activity for hydrogen evolution via support interactions. Nano Lett..

[16] Laursen A.B., Kegnæs S., Dahl S., Chorkendorff I. (2012). Molybdenum sulfides—Efficient and viable materials for electro-and photoelectrocatalytic hydrogen evolution. Energy Environ. Sci..

[17] Fan X.B., Xu P.T., Zhou D.K., Sun Y.F., Li Y.G.C., Nguyen M.A.T., Terrones M., Mallouk T.E. (2015). Fast and Efficient Preparation of Exfoliated 2H MoS_2_ Nanosheets by Sonication-Assisted Lithium Intercalation and Infrared Laser-Induced 1T to 2H Phase Reversion. Nano Lett..

[18] Jiang H., Ren D., Wang H., Hu Y., Guo S., Yuan H., Hu P., Zhang L., Li C. (2015). 2D monolayer MoS_2_–carbon interoverlapped superstructure: Engineering ideal atomic interface for lithium ion storage. Adv. Mater..

[19] Xie J., Zhang J., Li S., Grote F., Zhang X., Zhang H., Wang R., Lei Y., Pan B., Xie Y. (2013). Controllable disorder engineering in oxygen-incorporated MoS_2_ ultrathin nanosheets for efficient hydrogen evolution. J. Am. Chem. Soc..

[20] Chen B.A., Lu H.H., Zhou J.W., Ye C., Shi C.S., Zhao N.Q., Qiao S.Z. (2018). Porous MoS_2_/Carbon Spheres Anchored on 3D Interconnected Multiwall Carbon Nanotube Networks for Ultrafast Na Storage. Adv. Energy Mater..

[21] Joyner J., Oliveira E.F., Yamaguchi H., Kato K., Vinod S., Galvao D.S., Salpekar D., Roy S., Martinez U., Tiwary C.S. (2020). Graphene Supported MoS_2_ Structures with High Defect Density for an Efficient HER Electrocatalysts. Acs Appl. Mater. Interfaces.

[22] Lin J.H., Wang P.C., Wang H.H., Li C., Si X.Q., Qi J.L., Cao J., Zhong Z.X., Fei W.D., Feng J.C. (2019). Defect-Rich Heterogeneous MoS_2_/NiS_2_ Nanosheets Electrocatalysts for Efficient Overall Water Splitting. Adv. Sci..

[23] Wu C.L., Huang P.C., Brahma S., Huang J.L., Wang S.C. (2017). MoS_2_-MoO_2_ composite electrocatalysts by hot-injection method for hydrogen evolution reaction. Ceram. Int.

[24] Qiao S.L., Zhang B.Y., Li Q., Li Z., Wang W.B., Zhao J., Zhang X.J., Hu Y.Q. (2019). Pore Surface Engineering of Covalent Triazine Frameworks@MoS_2_ Electrocatalyst for the Hydrogen Evolution Reaction. Chemsuschem.

[25] Ding Q., Song B., Xu P., Jin S. (2016). Efficient Electrocatalytic and Photoelectrochemical Hydrogen Generation Using MoS_2_ and Related Compounds. Chem-Us.

[26] Wang Y.N., Zhang F.F., Yang M.K., Wang Z., Ren Y.Y., Cui J., Zhao Y.G., Du J.M., Li K.D., Wang W.M. (2019). Synthesis of porous MoS_2_/CdSe/TiO_2_ photoanodes for photoelectrochemical water splitting. Microporous Microporous Mater..

[27] Chee S.S., Seo D., Kim H., Jang H., Lee S., Moon S.P., Lee K.H., Kim S.W., Choi H., Ham M.H. (2019). Lowering the Schottky Barrier Height by Graphene/Ag Electrodes for High-Mobility MoS_2_ Field-Effect Transistors. Adv. Mater..

[28] Xu J., Shim J., Park J.H., Lee S. (2016). MXene Electrode for the Integration of WSe_2_ and MoS_2_ Field Effect Transistors. Adv. Funct. Mater..

[29] Li D.J., Maiti U.N., Lim J., Choi D.S., Lee W.J., Oh Y., Lee G.Y., Kim S.O. (2014). Molybdenum Sulfide/N-Doped CNT Forest Hybrid Catalysts for High-Performance Hydrogen Evolution Reaction. Nano Lett..

[30] Shi Y., Wang Y., Wong J.I., Tan A.Y.S., Hsu C.-L., Li L.-J., Lu Y.-C., Yang H.Y. (2013). Self-assembly of hierarchical MoSx/CNT nanocomposites (2 < x < 3): Towards high performance anode materials for lithium ion batteries. Sci. Rep..

[31] Hu W.-H., Yu R., Han G.-Q., Liu Y.-R., Dong B., Chai Y.-M., Liu Y.-Q., Liu C.-G. (2015). Facile synthesis of MoS_2_/RGO in dimethyl-formamide solvent as highly efficient catalyst for hydrogen evolution. Mater. Lett..

[32] Zhou J., Qin J., Zhang X., Shi C., Liu E., Li J., Zhao N., He C. (2015). 2D space-confined synthesis of few-layer MoS_2_ anchored on carbon nanosheet for lithium-ion battery anode. Acs Nano.

[33] Xie X., Ao Z., Su D., Zhang J., Wang G. (2015). MoS_2_/Graphene Composite Anodes with Enhanced Performance for Sodium-Ion Batteries: The Role of the Two-Dimensional Heterointerface. Adv. Funct. Mater..

[34] Chang K., Chen W., Ma L., Li H., Li H., Huang F., Xu Z., Zhang Q., Lee J.-Y. (2011). Graphene-like MoS_2_/amorphous carbon composites with high capacity and excellent stability as anode materials for lithium ion batteries. J. Mater. Chem..

[35] Toby B.H., Von Dreele R.B. (2013). GSAS-II: The genesis of a modern open-source all purpose crystallography software package. J. Appl Crystallogr..

[36] Chen S., Ju Y.Y., Guo Y., Xiong C.X., Dong L.J. (2017). In-site synthesis of monodisperse, oleylamine-capped Ag nanoparticles through microemulsion approach. J. Nanopart Res..

[37] Pan Y., Bai H.Y., Pan L., Li Y.D., Tamargo M.C., Sohel M., Lombardi J.R. (2012). Size controlled synthesis of monodisperse PbTe quantum dots: Using oleylamine as the capping ligand. J. Mater. Chem..

[38] Vinoth R., Patil I.M., Pandikumar A., Kakade B.A., Huang N.M., Dionysios D.D., Neppolian B. (2016). Synergistically Enhanced Electrocatalytic Performance of an N-Doped Graphene Quantum Dot-Decorated 3D MoS_2_-Graphene Nanohybrid for Oxygen Reduction Reaction. Acs Omega.

[39] Yi M.R., Zhang C.H. (2018). The synthesis of two-dimensional MoS_2_ nanosheets with enhanced tribological properties as oil additives. Rsc Adv..

[40] Shi Z.T., Kang W.P., Xu J., Sun Y.W., Jiang M., Ng T.W., Xue H.T., Yu D.Y.W., Zhang W.J., Lee C.S. (2016). Hierarchical nanotubes assembled from MoS_2_-carbon monolayer sandwiched superstructure nanosheets for high-performance sodium ion batteries. Nano Energy.

[41] Sha J.W., Gao C.T., Lee S.K., Li Y.L., Zhao N.Q., Tour J.M. (2016). Preparation of Three-Dimensional Graphene Foams Using Powder Metallurgy Templates. Acs Nano.

[42] Stankovich S., Dikin D.A., Piner R.D., Kohlhaas K.A., Kleinhammes A., Jia Y., Wu Y., Nguyen S.T., Ruoff R.S. (2007). Synthesis of graphene-based nanosheets via chemical reduction of exfoliated graphite oxide. Carbon.

[43] Zheng X., Xu J., Yan K., Wang H., Wang Z., Yang S. (2014). Space-confined growth of MoS_2_ nanosheets within graphite: The layered hybrid of MoS_2_ and graphene as an active catalyst for hydrogen evolution reaction. Chem. Mater..

[44] Li W., Bi R., Liu G.X., Tian Y.X., Zhang L. (2018). 3D Interconnected MoS_2_ with Enlarged Interlayer Spacing Grown on Carbon Nanofibers as a Flexible Anode Toward Superior Sodium-Ion Batteries. Acs Appl. Mater. Interfaces.

[45] Freeman T.L., Evans S.D., Ulman A. (1995). Xps Studies of Self-Assembled Multilayer Films. Langmuir.

[46] Dong Y.R., Jiang H., Deng Z.N., Hu Y.J., Li C.Z. (2018). Synthesis and assembly of three-dimensional MoS_2_/rGO nanovesicles for high-performance lithium storage. Chem Eng. J..

[47] Koroteev V.O., Bulusheva L.G., Asanov I.P., Shlyakhova E.V., Vyalikh D.V., Okotrub A.V. (2011). Charge Transfer in the MoS_2_/Carbon Nanotube Composite. J. Phys. Chem C.

[48] Hong J., Hu Z., Probert M., Li K., Lv D., Yang X., Gu L., Mao N., Feng Q., Xie L. (2015). Exploring atomic defects in molybdenum disulphide monolayers. Nat. Commun..

[49] Li G.Q., Zhang D., Qiao Q., Yu Y.F., Peterson D., Zafar A., Kumar R., Curtarolo S., Hunte F., Shannon S. (2016). All The Catalytic Active Sites of MoS_2_ for Hydrogen Evolution. J. Am. Chem. Soc..

[50] Li H., Tsai C., Koh A.L., Cai L.L., Contryman A.W., Fragapane A.H., Zhao J.H., Han H.S., Manoharan H.C., Abild-Pedersen F. (2016). Activating and optimizing MoS_2_ basal planes for hydrogen evolution through the formation of strained sulphur vacancies. Nat. Mater..

[51] Wang Y., Yang Y., Zhang D., Wang Y., Luo X., Liu X., Kim J.-K., Luo Y. (2020). Inter-overlapped MoS_2_/C composites with large-interlayer-spacing for high-performance sodium-ion batteries. Nanoscale Horiz..

[52] Li Y.G., Wang H.L., Xie L.M., Liang Y.Y., Hong G.S., Dai H.J. (2011). MoS_2_ Nanoparticles Grown on Graphene: An Advanced Catalyst for the Hydrogen Evolution Reaction. J. Am. Chem. Soc..

[53] Wang H., Xiao X., Liu S.Y., Chiang C.L., Kuai X.X., Peng C.K., Lin Y.C., Meng X., Zhao J.Q., Choi J.H. (2019). Structural and Electronic Optimization of MoS_2_ Edges for Hydrogen Evolution. J. Am. Chem. Soc..

[54] Yan Y., Ge X., Liu Z., Wang J.-Y., Lee J.-M., Wang X. (2013). Facile synthesis of low crystalline MoS_2_ nanosheet-coated CNTs for enhanced hydrogen evolution reaction. Nanoscale.

[55] Guo Y., Zhang X., Zhang X., You T. (2015). Defect-and S-rich ultrathin MoS_2_ nanosheet embedded N-doped carbon nanofibers for efficient hydrogen evolution. J. Mater. Chem. A.

[56] Chen T.-Y., Chang Y.-H., Hsu C.-L., Wei K.-H., Chiang C.-Y., Li L.-J. (2013). Comparative study on MoS_2_ and WS_2_ for electrocatalytic water splitting. Int. J. Hydrog. Energy.

